# Nasopharyngeal carcinoma: epidemiology, risk factors, signaling pathways, clinical features, diagnosis, and management

**DOI:** 10.1186/s43556-026-00590-6

**Published:** 2026-09-17

**Authors:** Yangbingyu Wen, Yue Xu, Yao Du, Yuting Zhan, Songqing Fan

**Affiliations:** 1 Department of Pathology, The Second Xiangya Hospital, Central South University, Changsha, Hunan 410011 China; 2 Hunan Clinical Medical Research Center for Cancer Pathogenic Genes Testing and Diagnosis, Changsha, Hunan 410011 China

**Keywords:** Nasopharyngeal carcinoma, Epstein–Barr virus, Molecular pathogenesis, Signaling pathways, Plasma EBV DNA, Immunotherapy

## Abstract

Nasopharyngeal carcinoma (NPC) is a distinctive epithelial malignancy characterized by marked geographic and ethnic clustering, with the greatest burden concentrated in East and Southeast Asia despite declining age-standardized incidence and mortality rates in many regions. NPC arises from a complex interaction among persistent Epstein–Barr virus (EBV) infection, inherited susceptibility, and environmental exposures. Among these factors, EBV plays a central role in endemic NPC through latent gene products, particularly latent membrane protein 1 (LMP1), latent membrane protein 2A (LMP2A), and Epstein–Barr nuclear antigen 1 (EBNA1), which sustain oncogenic signaling, remodel the tumor microenvironment, and reinforce malignant progression. Clinically, NPC is often diagnosed at a locoregionally advanced stage because of its deep anatomical location and nonspecific early symptoms, making accurate detection, risk stratification, and treatment selection particularly important. Although previous reviews have addressed individual aspects of NPC biology or management, an integrated framework linking epidemiological determinants, viral oncogenesis, host molecular dysregulation, and therapeutic evolution remains needed. This review therefore synthesizes current evidence on NPC epidemiology, risk factors, EBV-associated molecular mechanisms, major signaling pathways and their crosstalk, genetic and epigenetic alterations, clinical features, diagnosis, staging, and management. Particular emphasis is placed on the clinical roles and limitations of plasma EBV DNA, the distinction between established standards and investigational strategies, and emerging molecular and immune-based therapies. By linking mechanistic insights with their clinical and translational implications, this review provides a biologically informed framework for understanding NPC and highlights priorities for biomarker standardization, risk-adapted treatment, and future therapeutic development.

## Introduction

Nasopharyngeal carcinoma (NPC) is a distinctive epithelial malignancy with unique etiological, molecular and clinical features [1]. According to the fifth edition of the WHO Classification of Head and Neck Tumors, NPC includes keratinizing squamous cell carcinoma, non-keratinizing carcinoma with differentiated and undifferentiated forms, and the rare basaloid squamous cell carcinoma [2]. These subtypes differ in geographic distribution, Epstein–Barr virus (EBV) association and clinical behavior. NPC subtypes show strong geographic variation: non‑keratinizing NPC, tightly linked to EBV, predominates in high‑incidence regions; conversely, keratinizing NPC is more common in low‑incidence areas, is less consistently EBV‑positive, and generally carries a worse prognosis [3, 4].

Unlike other cancers, NPC is closely linked to Epstein–Barr virus (EBV), shows a striking geographic and ethnic predilection, and is characterized by a complex interplay between viral infection, host susceptibility and environmental exposure [5–9]. These features make NPC not only a clinically important disease, but also a biologically informative model for studying virus-associated carcinogenesis. Among these factors, EBV occupies a central position, especially in endemic non-keratinizing NPC [1]. EBV is maintained in a type II latency program in NPC cells, permitting sustained expression of latent viral products that support tumor initiation and progression [5]. At the same time, host susceptibility loci, particularly within the HLA region, indicate that interindividual differences in immune recognition of viral antigens are likely to influence disease risk [10, 11]. Environmental exposures, including Chinese-style salted fish and tobacco smoking, further modify this background risk and may help explain both endemic clustering and temporal variation in incidence [9, 12, 13]. Thus, NPC is not simply an EBV-associated cancer, but a malignancy that arises through the cumulative interaction of viral persistence, host biology and exposure history.

Over the past decade, substantial progress has also been made in understanding NPC beyond classical descriptive clinicopathological frameworks. Genomic and epigenomic studies have clarified that NPC is characterized by selective somatic lesions [14], widespread DNA hypermethylation [15], enhancer reprogramming [16], and chromatin remodeling [17]. In parallel, advances in MRI [18], plasma EBV DNA analysis, and radiotherapy [19] have improved detection, staging, and disease control, while induction chemotherapy [20], adjuvant chemotherapy [21] and PD-1 inhibitor [22] have expanded the therapeutic landscape for both locoregionally advanced and recurrent/metastatic disease.

In this review, we provide an integrated overview of NPC, covering epidemiology, risk factors and etiology, molecular basis and signaling pathways, clinical features, diagnosis and staging, and management. The discussion of molecular pathogenesis focuses mainly on non-keratinizing, EBV-associated NPC, which represents the predominant subtype in endemic regions. Rather than presenting these topics as separate domains, we aim to highlight how endemic distribution, viral oncogenesis, inherited susceptibility, environmental exposure, host molecular dysregulation and therapeutic evolution are biologically and clinically connected. This review also allows us to identify current gaps, including the need for standardized EBV DNA testing, improved biologically informed risk stratification, more precise treatment selection, and better integration of emerging molecular and immune-based therapeutic strategies.

## Epidemiology

NPC is a relatively rare malignancy worldwide, with approximately 120,000 new cases diagnosed in 2022, but it exhibits one of the most pronounced geographic and ethnic disparities among human cancers [23], with the disease burden concentrated predominantly in East and Southeast Asia and particularly in China [24]. Clear regional differences are also seen within China, where the incidence in southern populations is substantially higher than that in northern populations [25, 26].

Migration studies further support the contribution of population background to the distinctive distribution of NPC. In a large Israeli cohort, individuals of North African or Asian origin showed an elevated risk of NPC, and this increased susceptibility persisted among second- and third-generation descendants [27]. A pronounced sex disparity is also evident, with age-standardized incidence and mortality rates in men approximately threefold higher than those in women [24].

The global burden of NPC is also changing over time. Over recent decades, age-standardized incidence and mortality rates have declined in many historically high-incidence and low-incidence regions [28]. However, declining rates do not necessarily translate into a lower absolute disease burden. The annual numbers of NPC cases and deaths are projected to reach approximately 179,476 and 113,851, respectively, by 2040 [24], reflecting the effects of population growth and aging. Together, these pronounced geographic, ethnic, sex, and migration-related disparities suggest that the global distribution of NPC reflects the combined influence of population susceptibility and environmental context. These epidemiological patterns provide the basis for examining the viral, genetic, and environmental determinants of NPC risk.

## Risk factors

**Fig. 1:**
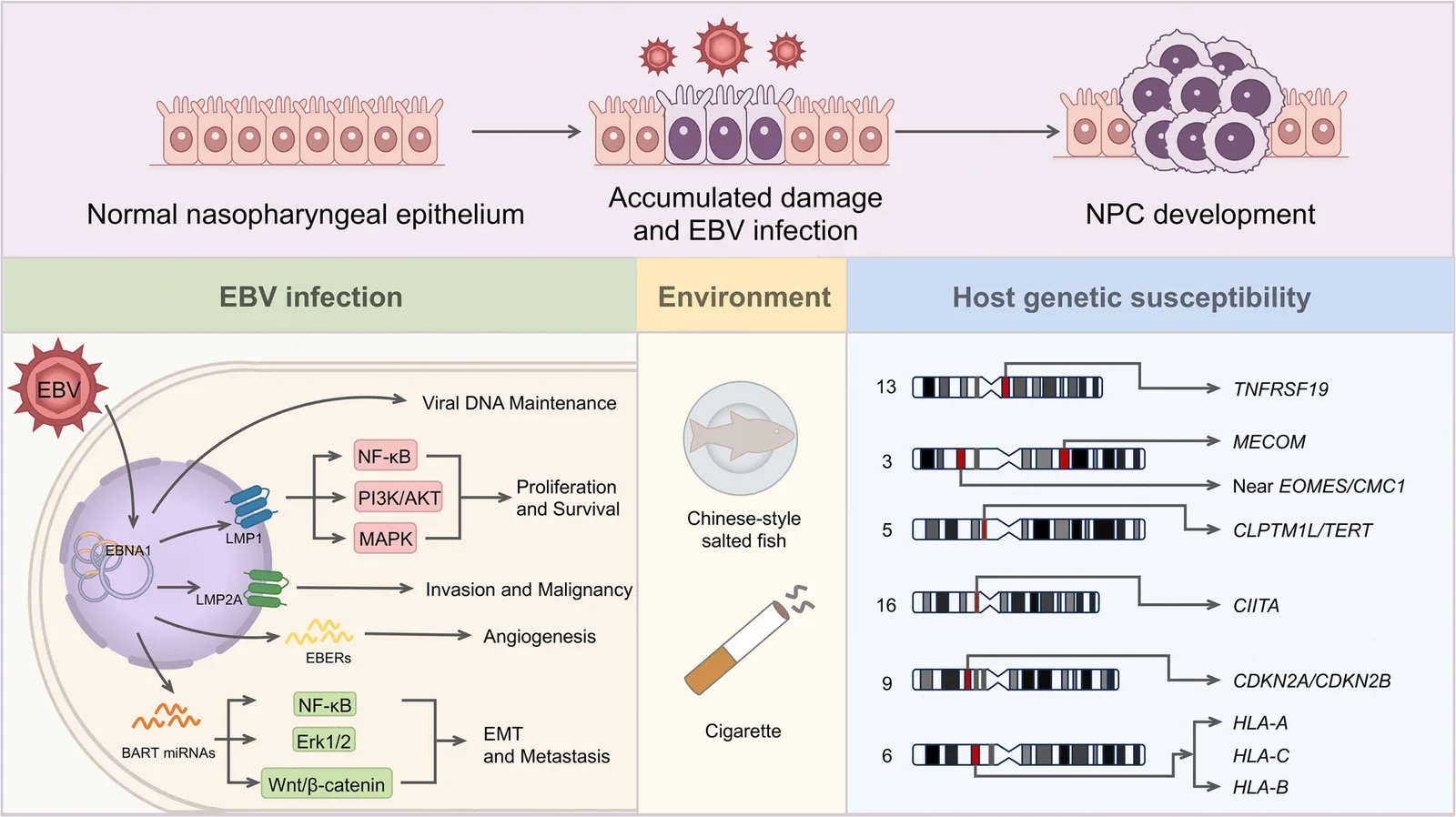
Multifactorial pathogenesis in NPC. Nasopharyngeal carcinoma develops through the combined effects of Epstein–Barr virus infection, environmental exposure and host genetic susceptibility. EBV latent products contribute to viral genome maintenance, oncogenic signaling, epithelial–mesenchymal transition, angiogenesis and metastasis. Environmental factors, including Chinese-style salted fish and cigarette smoking, may further modify disease risk. Host susceptibility loci, particularly within the human leukocyte antigen region and other genome-wide association study-identified loci, help explain interindividual and population-level differences in NPC risk. Abbreviations: BART miRNAs, BamHI-A rightward transcript microRNAs; EBERs, Epstein–Barr virus-encoded small RNAs; EBNA1, Epstein–Barr nuclear antigen 1; EBV, Epstein–Barr virus; EMT, epithelial–mesenchymal transition; ERK1/2, extracellular signal-regulated kinase 1/2; HLA, human leukocyte antigen; LMP1, latent membrane protein 1; LMP2A, latent membrane protein 2A; MAPK, mitogen-activated protein kinase; NF-κB, nuclear factor kappa B; NPC, nasopharyngeal carcinoma; PI3K/AKT, phosphoinositide 3-kinase/protein kinase B

The distinctive epidemiological distribution of NPC reflects the combined influence of viral, genetic, and environmental determinants [1]. Among these, EBV is the central etiological factor in endemic non-keratinizing NPC, but EBV infection alone is not sufficient to explain disease development, given its near-ubiquitous distribution in the general population [5, 29]. Current evidence suggests that host genetic background may shape individual susceptibility to EBV-associated tumorigenesis, whereas environmental exposures further modify risk across different populations and life stages [11, 12]. Therefore, these factors help explain the distinctive geographic distribution, endemic clustering, and marked inter-individual heterogeneity (Fig. 1).

### EBV infection

Epstein-Barr virus (EBV) is closely associated with the non-keratinizing subtype of NPC, which predominates in endemic regions. Previous studies have shown that EBV genomes predominantly exist as clonal episomes in NPC tumor cells [5, 30]. This supports the view that EBV infection occurs before or during early clonal expansion and represents an early event in NPC development.

In NPC, EBV is maintained in type II latency, in which Epstein–Barr nuclear antigen 1 (EBNA1), latent membrane proteins 1 and 2A (LMP1 and LMP2A), and EBV-encoded non-coding RNAs are expressed. EBV promotes NPC development through the coordinated actions of these genes. EBNA1, the only viral protein consistently expressed in all EBV-positive tumors [31], is essential for the maintenance of latent EBV infection [32]. By binding to the viral origin of plasmid replication (OriP), and interacting with the family of repeats (FR) region and host chromosomal DNA, EBNA1 ensures stable replication and segregation of the episomal EBV genome during host cell division [33]. This stable latent state enables continued expression of viral products that can influence host-cell behavior.

LMP1 and LMP2A are commonly detected in NPC and play important roles in tumor progression [34]. LMP1 acts as a signaling driver by activating pathways such as NF-κB [35], PI3K/AKT [36], and MAPK [37], thereby promoting proliferation, survival, and inflammatory signaling. LMP2A has been implicated in enhanced cell survival, invasive behavior, and maintenance of the malignant phenotype [38]. In addition, EBV-encoded non-coding RNAs also contribute to NPC progression. EBERs have been reported to sustain latent infection [39], promote RIG-I-mediated inflammatory signaling [40] and enhance angiogenesis [41]. BART miRNAs, although functionally heterogeneous, have also been implicated in epithelial-mesenchymal transition and metastatic potential through activating pathways such as NF-κB [42], Erk1/2 [43], and Wnt/β-catenin [44].

Taken together, these findings support a central role for EBV in NPC pathogenesis. By maintaining latent persistence and enabling continued expression of viral products, EBV may exert a sustained influence on immune responses and tumor-associated behavior.

### Host genetic susceptibility

Host genetic factors are thought to shape the susceptible background of NPC rather than acting as independent oncogenic drivers. Epidemiological and genetic evidence indicates that, despite the high prevalence of EBV infection in the general population, only a small fraction of infected individuals, especially those exhibiting high-risk serological characteristics, progress to NPC [45, 46]. Therefore, genetic background may modulate individual responses to viral infection and environmental exposures, thereby influencing disease susceptibility.

Evidence from both candidate gene studies and genome-wide association studies (GWAS) has identified variants in major histocompatibility complex (MHC) class I genes on chromosome 6p21.3 as major susceptibility loci for NPC, particularly HLA [6, 47]. The MHC class I genes *HLA-A*, *HLA-B* and *HLA-C* encode proteins that mediate antigen presentation to cytotoxic T cells and are central to the recognition of EBV-derived antigens. Natural polymorphisms in HLA genes can alter the peptide-binding repertoire and antigen-presenting characteristics of MHC class I molecules, which may modulate susceptibility to NPC [48]. Studies have reported that carriers of HLA-A*02:07, HLA-A*33:03, and HLA-B*38:02 have an increased risk of NPC, whereas HLA-A11:01, HLA-B13:01, and HLA-B55:02 are associated with reduced susceptibility [10, 11].Accordingly, inherited variation within these loci may influence peptide-binding preferences and antigen-presentation efficiency, thereby contributing to inter-individual differences in NPC susceptibility.

Beyond the MHC region, GWAS have reported additional NPC susceptibility loci, including 13q12 (*TNFRSF19*), 3q26 (*MECOM*), 5p15 (*CLPTM1L/TERT*), 16p13 (*CIITA*), 9p21 (*CDKN2A/CDKN2B*), and 3p24.1 (near *EOMES/CMC1*) [7, 49–51]. Among them, TNFRSF19 and MECOM are supported by relatively direct functional studies in NPC, while emerging evidence supports a functional role for EOMES and CLPTM1L. Although the evidence for these loci is less consistent than that for HLA, they suggest that host susceptibility to NPC may extend beyond antigen presentation alone. These genes point to additional pathways that may shape the susceptible background of NPC, including immune regulation, cell cycle control, telomere maintenance, and tumor progression.

Overall, host genetic factors are unlikely to act as independent causes of NPC but instead define a susceptible background in which EBV infection and environmental exposures are more likely to promote tumor development. HLA-mediated antigen recognition appears to represent a core of this inherited susceptibility. In addition, other loci imply that inherited susceptibility may involve broader processes that influence the immune response to EBV infection and other carcinogenic exposures.

### Environmental factors

Environmental exposures have been consistently implicated in NPC, particularly in relation to its characteristic geographic clustering, although the strength of evidence varies across exposure categories. Among these, dietary factors have been most extensively studied in endemic populations. In particular, Chinese-style salted fish has long been regarded as a classical environmental risk factor for NPC, with early case–control studies suggesting that the association is strongest for exposure during childhood [8, 52]. However, more recent large population-based studies suggest that the association may be weaker than previously estimated, particularly for adult intake. In a case–control study including 2554 cases and 2648 controls from southern China, most associations were moderate, and weekly salted-fish intake at age 10 was associated with an odds ratio (OR) of 1.56 (95% CI 1.24–1.97), substantially lower than earlier reports [12]. This apparent attenuation may reflect differences in exposure patterns, study design, and confounding control across populations, and suggests that the contribution of salted fish is likely to depend on the timing, frequency, and type of exposure rather than being a uniformly strong effect.

Smoking is another relatively consistent risk factor. Prospective data from endemic areas have demonstrated a dose-dependent relationship between tobacco exposure and NPC risk [9], and case–control studies likewise support positive associations for both active and passive smoking, although the magnitude of risk varies across studies [13]. Whether smoking contributes to NPC risk partly through effects on EBV-related processes remains uncertain. Some studies have reported associations between smoking and elevated anti-EBV IgA antibody levels, which may reflect altered virus–host interactions [53–55]. However, other studies in high-risk populations have not observed consistent associations with EBV DNA load or serological markers [56]. Taken together, these findings support an epidemiological role for smoking in NPC while indicating that the underlying biological mechanisms, including potential links to EBV dynamics, remain incompletely defined.

Other environmental exposures, including additional preserved foods [52] and selected occupational or inhalational exposures [57, 58], have also been investigated, although the available evidence is generally less consistent and more heterogeneous, reflecting differences in study design, exposure assessment, and population context. Overall, current evidence suggests that environmental exposures are risk factors, with effects that are shaped by exposure timing and host background rather than functioning as uniformly strong or independent determinants.

## Molecular basis and signaling pathways

The risk factors described above can affect NPC development through changes in viral and host cell functions. In EBV-associated NPC, viral latent products act together with genetic and epigenetic alterations to affect several signaling pathways involved in cell survival, proliferation, invasion, immune regulation, and treatment response [5, 14–17, 35]. These pathways include NF-κB, PI3K/AKT/mTOR, JAK/STAT, MAPK, Wnt/β-catenin, and hypoxia-related signaling. Their effects often overlap and contribute to common malignant features of NPC.

### EBV oncoproteins in NPC pathogenesis

Among the EBV latent proteins expressed in NPC, LMP1, LMP2A, and EBNA1 have different but partly overlapping functions [34]. LMP1 acts as a constitutively active signaling regulator, whereas LMP2A affects cell survival, epithelial plasticity, and invasive behavior [59]. EBNA1 is required for the stable maintenance of the episomal EBV genome and can also alter host cellular processes [32]. Together, these proteins contribute to several signaling abnormalities observed in NPC (Table 1).

**Table 1:** Major function of EBV oncoproteins in nasopharyngeal carcinoma

Protein	Downstream effectors/target	Function	Ref
LMP1	PD-L1	Upregulates PD-L1 expression	[60]
	ALIX	Mediates immune escape	[61]
	NF-κB/IL-8	Promotes IL-8 production and angiogenesis	[62, 63]
	GLUT1	Reprograms glycolysis	[64]
	PKCδ	Drives malignant progression	[65]
	MEK/ERK	Promotes EMT and proliferation	[66, 67]
	E-cadherin	Promotes E-cadherin silencing and cell migration	[68]
	HIF-1α	Promotes angiogenesis	[69]
	PI3K/AKT	Promotes proliferation, survival and migration; suppresses apoptosis	[36, 70]
	NTRK2	Promotes EMT, anoikis resistance and metastasis	[71]
	TGF-β	Promotes EMT, migration and invasion	[72]
	Twist	Promotes EMT	[73]
	Snail	Promotes EMT	[74]
	BNIP3	Promotes protective autophagy and radioresistance	[75]
	mTORC1/2	Imposes stem-like characteristics and chemoresistance	[76]
	PGC-1α	Promotes anoikis resistance and immune escape	[77]
LMP2A	SYK/ITGβ4	Promotes migration and invasion	[38]
	EGFR	Promotes cell migration	[78]
	ERK	Promotes stem-like characteristics; promotes invasion	[79, 80]
	Hedgehog pathway	Promotes stem-like characteristics	[81]
	WNT5A	Promotes proliferation, migration and invasion	[82]
EBNA1	OriP/FR and host chromosomes	Maintains replication and segregation of the EBV episome	[32, 33]
	PML nuclear bodies	Impairs DNA repair, p53 activation and apoptosis	[83]
	c-Jun	Promotes angiogenesis	[84]
	ZEB1/2	Promotes EMT, migration and invasion	[85]
	CDC7/POU2F1	Promotes cell-cycle progression and tumor-cell growth	[86]
	TGF-β1	Promotes Treg accumulation	[87]
	PI3K/AKT	Promotes Treg chemoattraction	[88]

*Abbreviations*: *LMP1* latent membrane protein 1, *NF-κB* nuclear factor kappa B, *JAK/STAT* Janus kinase/signal transducer and activator of transcription, *MEK/ERK* mitogen-activated protein kinase kinase/extracellular signal-regulated kinase, *PI3K/AKT* phosphoinositide 3-kinase/protein kinase B, *HIF-1α* hypoxia-inducible factor 1 alpha, *TGF-β* transforming growth factor beta, *IL-8* interleukin 8, *ALIX* ALG-2-interacting protein X, *GLUT1* glucose transporter 1, *BNIP3* BCL2 interacting protein 3, *mTORC1/mTORC2* mechanistic target of rapamycin complex 1/2, *PGC-1α* peroxisome proliferator-activated receptor gamma coactivator 1 alpha

#### LMP1

LMP1 is a major EBV oncoprotein with broad effects on host cell signaling. It functions as a constitutively active receptor-like protein and shares functional properties with CD40, a member of the tumor necrosis factor receptor family [59]. Its cytoplasmic CTAR1 and CTAR2 domains recruit downstream signaling proteins and allow persistent activation of several pathways involved in NPC progression [59].

LMP1 can activate several oncogenic signaling pathways in NPC, among which its association with NF-κB is the most consistently established. Experimental studies in NPC cells have shown that LMP1 promotes IκBα degradation and NF-κB activation [89]. Genomic studies further suggest that LMP1 expression and somatic alterations affecting negative regulators of NF-κB can produce similar changes in this pathway, supporting an important role for sustained NF-κB activity in inflammation and immune escape [35].

LMP1 also contributes to immune regulation in NPC and has been shown to upregulate PD-L1 expression in nasopharyngeal epithelial and NPC cells, supporting a role for EBV-associated signaling in immune escape [60]. LMP1 also promotes the secretion of PD-L1-containing small extracellular vesicles through ALIX [61]. Disruption of this process reduced immunosuppressive vesicle secretion and improved CD8 + T-cell responses in experimental models [61]. LMP1 also affects the tumor microenvironment (TME). It can induce IL-8 expression through NF-κB signaling and has been associated with increased angiogenesis in NPC [62, 63]. In addition, LMP1 can increase the expression of inflammatory cytokines and chemokines that influence immune-cell recruitment and communication within the tumor microenvironment [90]. Beyond TME remodeling, LMP1 can also promote glycolytic reprogramming in NPC cells, increasing glucose uptake and lactate production [64].

#### LMP2A

LMP2A contributes to NPC progression by promoting cell migration, invasion, epithelial plasticity, and stem-like properties. Early studies showed that LMP2A enhances epithelial cell migration and invasion [91, 92]. Mechanistically, LMP2A can disrupt the interaction between spleen tyrosine kinase (SYK) and integrin β4 (ITGβ4) by competitively binding to SYK, thereby altering the localization and function of ITGβ4 and promoting cell motility [38]. A complementary study further showed that LMP2A enhances EGFR activation and intracellular Ca^2^⁺ signaling, leading to calpain-dependent cleavage and redistribution of ITGβ4 and increased NPC cell migration [78].

LMP2A also regulates several signaling pathways involved in invasive and stem-like phenotypes. It can increase MMP9 expression through ERK1/2-dependent induction of Fra-1, thereby promoting NPC cell invasion [79]. ERK activation mediated by LMP2A has also been implicated in the maintenance of stem-like properties in NPC cells [80].

#### EBNA1

EBNA1 is essential for the maintenance and stable persistence of the episomal EBV genome and is consistently expressed in EBV-positive NPC. Beyond its role in viral persistence, EBNA1 can directly affect host cellular processes involved in tumor progression. EBNA1 can disrupt PML nuclear bodies through interaction with and degradation of PML proteins, thereby impairing DNA repair, p53 activation, and apoptosis and promoting the survival of cells with DNA damage [83]. EBNA1 can also bind to the promoters of c-Jun and ATF2 and increase their expression, thereby enhancing AP-1 transcriptional activity and angiogenic activity in vitro [84]. In addition, EBNA1 can promote epithelial–mesenchymal transition and enhance the migratory and invasive properties of NPC cells [85].

EBNA1 also regulates host genes involved in cell-cycle progression and stem-like properties. Recent work identified CDC7 and POU2F1 as direct functional targets of EBNA1 in EBV-positive epithelial tumor models, including NPC-derived models [86]. EBNA1 binding to these loci promotes their transcription, while pharmacological inhibition of EBNA1 DNA binding suppresses cell-cycle progression and tumor-cell growth [86].

Beyond these tumor-intrinsic effects, EBNA1 can also shape the immune microenvironment of NPC. It can promote Treg accumulation through TGF-β1-mediated Treg differentiation, CCL20-dependent Treg migration, and M2 macrophage polarization [87]. A complementary mechanistic study further showed that EBNA1 promotes Treg chemoattraction through the TGFβ1–SMAD3–PI3K–AKT–c-JUN–miR-200a–CXCL12/CXCR4 signaling axis [88]. EBNA1 has also emerged as a potential therapeutic target. VK-2019, a small-molecule EBNA1 inhibitor, has entered first-in-human clinical evaluation in patients with EBV-positive NPC, although its therapeutic role remains investigational [93].

### Core dysregulated signaling pathways in NPC

**Fig. 2:**
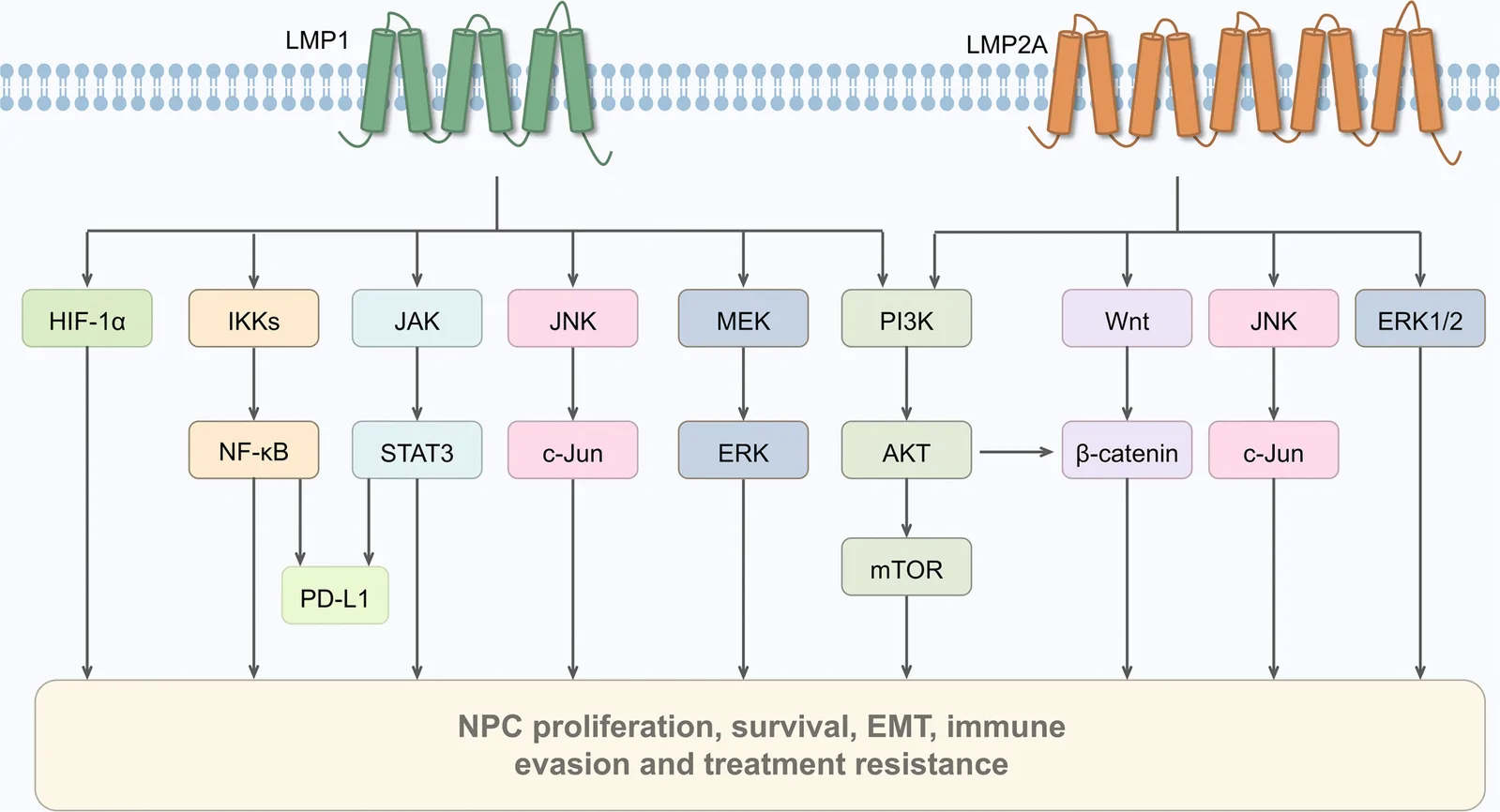
EBV-driven signaling crosstalk in NPC. The Epstein–Barr virus latent membrane proteins LMP1 and LMP2A engage multiple oncogenic signaling pathways in NPC. LMP1 activates NF-κB, JAK/STAT3, PI3K/AKT/mTOR, MEK/ERK, JNK/c-Jun, and HIF-1α signaling, whereas LMP2A contributes to PI3K/AKT/mTOR, Wnt/β-catenin, JNK/c-Jun, and ERK1/2 signaling. NF-κB and STAT3 signaling converge on PD-L1 regulation, while these signaling programs collectively promote NPC proliferation, survival, epithelial–mesenchymal transition, immune evasion, and treatment resistance. Abbreviations: AKT, protein kinase B; EBV, Epstein–Barr virus; EMT, epithelial–mesenchymal transition; ERK, extracellular signal-regulated kinase; HIF-1α, hypoxia-inducible factor 1 alpha; IKK, inhibitor of κB kinase; JAK, Janus kinase; JNK, c-Jun N-terminal kinase; LMP1, latent membrane protein 1; LMP2A, latent membrane protein 2A; MEK, mitogen-activated protein kinase kinase; mTOR, mechanistic target of rapamycin; NF-κB, nuclear factor kappa B; NPC, nasopharyngeal carcinoma; PD-L1, programmed death-ligand 1; PI3K, phosphoinositide 3-kinase; STAT3, signal transducer and activator of transcription 3

The major signaling abnormalities in NPC do not operate as isolated pathways. Instead, viral oncoproteins, somatic genetic alterations, epigenetic dysregulation, and microenvironmental cues converge on interconnected signaling networks, particularly PI3K/AKT/mTOR, NF-κB, JAK/STAT, MAPK, and Wnt/β-catenin signaling. Crosstalk among these pathways reinforces malignant phenotypes, including sustained proliferation, epithelial plasticity, immune evasion, metabolic adaptation, and treatment resistance (Fig. 2).

#### PI3K/AKT/mTOR

Among the signaling networks dysregulated in NPC, the PI3K/Akt/mTOR cascade plays a pivotal role by coordinating cell proliferation, survival, and therapeutic resistance. In NPC cells, activation of this pathway supports sustained growth primarily through promotion of cell-cycle progression and suppression of apoptosis. FKHR and BAD are among the downstream effectors of AKT [94]. Phosphorylation of FKHR facilitates the G1/S transition and S-phase entry, thereby promoting cell proliferation through the release of cell-cycle constraints [95]. By contrast, phosphorylated BAD loses its ability to interact with Bcl-xL at the mitochondrial membrane, resulting in suppression of apoptosis [96]. Consistent with these observations, PI3K inhibitor LY294002 suppresses NPC cell growth and increases apoptotic cell death [97].

Beyond these effects, PI3K/AKT/mTOR signaling intersects with viral and inflammatory signaling programs that contribute to NPC progression. LMP1 can promote glycolytic reprogramming through mTORC1- and NF-κB-dependent upregulation of GLUT1 [64], linking this pathway to the metabolic adaptation of EBV-positive NPC cells. In parallel, IL-8 can activate AKT signaling and promote EMT, migration, and invasion [98]. These findings illustrate how PI3K/AKT/mTOR signaling integrates viral oncogenic signals with metabolic and invasive programs rather than functioning as an isolated proliferative pathway.

The PI3K/AKT/mTOR pathway has also been implicated in treatment resistance, particularly radioresistance. FLI1/TIE1-dependent activation of PI3K/AKT enhances DNA damage repair and radioresistance in NPC models [99]. Conversely, C2orf40 overexpression and CENP-N knockdown increase radiosensitivity through suppression of PI3K/AKT/mTOR or AKT/mTOR signaling [100, 101]. EXO-miR-197-3p similarly enhances radiosensitivity by inhibiting the AKT/mTOR axis and HSPA5-mediated autophagy [102]. More recent work has linked USP10-mediated stabilization of VDAC1 to mTOR-dependent autophagy, radioresistance, and metastatic behavior in experimental NPC models [103]. Although these findings identify PI3K/AKT/mTOR signaling as a potential target for radiosensitization, the supporting evidence is derived predominantly from preclinical models, and pathway-directed treatment has not yet established a clinical role in NPC.

#### NF-κB

NF-κB signaling is constitutively activated in NPC through convergent viral and host genetic mechanisms. LMP1 represents a major viral activator, whereas recurrent somatic alterations affecting negative regulators of NF-κB provide an alternative route to sustained pathway activation [104, 105]. Notably, high LMP1 expression is largely mutually exclusive with certain NF-κB-activating somatic alterations, suggesting that viral and host genetic events may represent alternative routes converging on a shared oncogenic signaling output [105]. NF-κB signaling comprises canonical and non-canonical branches with distinct activation mechanisms [106]. In NPC, canonical NF-κB activation, mediated through NEMO-dependent IKK activation and RelA/p50 nuclear translocation, has been more directly linked to inflammatory and immune-regulatory processes [107], whereas the role of non-canonical signaling remains less uniformly defined despite emerging evidence of RELB/NFKB2 (p100/p52) pathway involvement in NPC [108].

A major consequence of sustained NF-κB activation is the establishment of a pro-inflammatory and immunoregulatory tumor microenvironment. NF-κB promotes the expression of cytokines and chemokines, including IL-8, CCL3, CCL4, IL-1α, and IL-1β, several of which are induced by LMP1-mediated signaling [62, 109, 110]. These mediators can influence leukocyte recruitment and stromal interactions, while NF-κB-associated cytokine networks may also contribute to an immunosuppressive microenvironment involving Treg cells and MDSCs [111, 112]. Together with genomic and transcriptomic evidence linking NF-κB pathway alterations to defective immune surveillance [35], these findings support a broader role for NF-κB in connecting EBV-driven inflammation with immune evasion in NPC.

NF-κB signaling also intersects with immune checkpoint regulation and other oncogenic pathways. In preclinical models, chemotherapy and radiotherapy can induce PD-L1 expression in NPC cells through NF-κB-dependent mechanisms [113, 114]. More recent work extends this mechanism to the tumor vasculature, showing that crosstalk between NF-κB and STAT3 signaling promotes PD-L1 expression in tumor vascular endothelial cells and contributes to immune escape [115]. This interaction provides a direct link between the NF-κB and JAK/STAT networks within the NPC microenvironment. Nevertheless, although these findings implicate NF-κB-associated immune regulation as a potential therapeutic target, the clinical value of pathway-directed intervention in NPC remains to be established.

#### JAK/STAT

In NPC, the JAK/STAT pathway is activated mainly by viral and cytokine-mediated inputs. Research on the JAK/STAT pathway mainly focuses on the JAK2/STAT3 axis. LMP1 represents a characteristic upstream driver, which induces STAT3 phosphorylation and nuclear translocation and may further reinforce STAT3 activity through coupling with EGFR signaling [65, 116]. IL-6 is another important upstream activator and has been linked to malignant phenotypes [117]. These two inputs are not entirely independent. Previous studies suggest that LMP1 can enhance IL-6 production, whereas IL-6/IL-6R signaling in turn sustains STAT3 activation and may reinforce LMP1-associated oncogenic signaling, thereby forming a feed-forward circuit that helps maintain persistent JAK/STAT3 activity in NPC [118]. Recent studies found that tumor-intrinsic regulators [119], RNA networks [120] and biomechanical cues may also reinforce this pathway, indicating that this pathway is maintained by convergent rather than isolated upstream mechanisms.

JAK2/STAT3 signaling is most consistently associated with tumor cell survival and proliferation in NPC [117]. Early studies identified constitutive activation of STAT3 in NPC tissues [121], and subsequent work has further supported a central role for the JAK2/STAT3 axis in maintaining proliferative and anti-apoptotic signaling [117, 122]. This pathway can be reinforced through multiple upstream mechanisms, including enhanced JAK2 expression and relief of SOCS-mediated negative regulation, thereby promoting NPC cell growth and progression [119, 123]. In addition, recent studies further indicate that JAK2/STAT3 activation is linked to metastatic behavior and therapy resistance, suggesting that its role in NPC extends beyond growth maintenance alone [119, 124].

#### MAPK/ERK

Aberrant activation of the MAPK/ERK pathway is frequently observed in NPC, where it serves as a key driver of proliferation, invasive behavior, and adaptive responses to treatment. In NPC, MAPK/ERK activation is closely linked to LMP1, representing one of the most prominent upstream drivers [125]. LMP1 has been shown to activate MAPK/ERK signaling through its CTAR1 domain, whereas LMP1-targeted interventions suppress MEK/ERK activity and inhibit NPC cell growth [66, 67]. In an LMP1-independent manner, this pathway may also be deregulated by mutation in its key regulatory molecules, including Erb-B3 receptor tyrosine kinase 3 (*ERBB3*), v-raf murine sarcoma viral oncogene homolog B1 (*BRAF1*), *FGFR2*, *FGFR3*, and neurofibromatosis 1 (*NF1*) [105, 126]. In addition, epigenetic dysregulation provides another route to ERK hyperactivation. DNTTIP1 can recruit HDAC1 to the DUSP2 promoter, thereby repressing DUSP2 and enhancing ERK signaling [127].

In NPC, MAPK/ERK signaling is primarily linked to proliferative maintenance and invasive progression. The inhibition of the MAPK/ERK pathway has been shown to suppress NPC cell growth [128]. In addition, MAPK/ERK signaling has also been implicated in LMP1-driven epithelial–mesenchymal transition (EMT) [66]. This pathway furthermore participates in treatment resistance, particularly in relation to radioresistance and chemoresistance. Preclinical studies indicate that ERK inhibition can enhance treatment sensitivity in NPC cells [129, 130]. For example, the MEK inhibitor trametinib showed preclinical activity in overcoming chemoresistance in NPC models, supporting its further clinical evaluation in NPC [131].

#### JNK/c-Jun

Beyond ERK, the JNK/c-Jun axis is another extensively studied branch of MAPK signaling in NPC. Current evidence suggests that the JNK pathway is abnormally activated and is associated with invasive behavior and tumor progression in NPC [132]. The activation of JNK/c-Jun is closely associated with latent EBV infection. Early studies demonstrated that LMP1 can directly activate JNK, and subsequent mechanistic research showed that this process is mediated predominantly through a TRAF6–TAK1/TAB1–JNKK1/2 signaling cascade, rather than through a simple recapitulation of the canonical TNFR/IL-1R framework [133, 134]. In addition to LMP1, LMP2A also mediates the activation of the JNK pathway [135]. Stress associated with irradiation and altered adhesion status can also engage JNK signaling in NPC [136]. Elevated Jun and c-Jun expression has been associated with advanced TNM stage and poorer survival outcomes in NPC [132]. Notably, JNK activation can induce DNA methyltransferase activity, thereby promoting DNA hypermethylation and downregulation of E-cadherin expression [68, 137]. JNK/c-Jun signaling has also been implicated in radioresistance. Experimental studies indicate that downregulation of c-Jun enhances radiosensitivity and is associated with cell-cycle arrest and increased apoptosis in resistant NPC cells [138].

#### Wnt/β-catenin

Wnt/β-catenin signaling is frequently dysregulated in NPC, and contributes to multiple malignant behaviors. Current evidence suggests that aberrant Wnt/β-catenin activation in NPC is frequently associated with epigenetic silencing of negative pathway regulators, whereas canonical activating mutations such as those affecting CTNNB1 appear to be relatively uncommon [139, 140]. Promoter CpG island hypermethylation of multiple negative regulators is frequently observed in NPC, leading to transcriptional silencing or downregulation and thereby relieving inhibitory constraints on Wnt/β-catenin signaling [140]. EBV infection can also activate Wnt signalling [82]. LMP2A activates PI3K/Akt signaling and induces the inhibitory phosphorylation of glycogen synthase kinase 3β (GSK3β), thereby promoting β-catenin stabilization and nuclear accumulation and enhancing β-catenin-dependent transcription [141].

Several components of the Wnt/β-catenin pathway, including Wnt ligands and β-catenin, have been reported to be aberrantly expressed in NPC tissues and to correlate with clinicopathological progression and/or adverse survival outcomes [142–144]. Functionally, its prominent role appears to be the promotion of invasive and metastatic phenotypes. Aberrant β-catenin expression is often accompanied by reduced E-cadherin expression, suggesting a close connection between Wnt/β-catenin activation and EMT in NPC [145]. Consistent with this, ZNF488 overexpression and PEDF loss have both been shown to enhance EMT and invasiveness through Wnt/β-catenin activation, whereas β-catenin silencing markedly suppresses migratory and invasive behavior in NPC [146–148]. Beyond this role, Wnt/β-catenin signaling also appears to contribute to the maintenance of stem-like properties in NPC. Wnt/β-catenin inhibitors have been shown to suppress the growth of treatment-refractory cancer stem cell (CSC) populations [149, 150]. In addition, more recent studies suggest that Wnt/β-catenin signaling may directly or indirectly regulate PD-L1 transcription, thereby contributing to immune evasion [151].

#### Hypoxia/HIF-1α signaling

Unlike other signaling pathways, hypoxia-related signaling is more appropriately viewed as a microenvironment-driven adaptive program in NPC. Rapid tumor growth and inadequate perfusion together create a hypoxic microenvironment that activates hypoxia-related signaling [152]. In NPC, current evidence is centered predominantly on the HIF-1α axis. Hypoxia has been shown to induce the expression of HIF-1α, CAIX and VEGF, and cause extensive upregulation and downregulation of gene expression in a variety of biological processes [153]. In NPC tissues, HIF-1α and its downstream hypoxia marker CAIX are frequently upregulated and have been associated with adverse clinical outcomes [154]. Earlier studies likewise documented coordinated expression of HIF-1α, CAIX and VEGF, with concurrent high HIF-1α and CAIX expression identifying patients with more aggressive disease behavior [155]. The activation of this signaling can also be regulated by EBV. On the one hand, LMP1 prolongs the half-life of HIF-1α mRNA by downregulating TTP and PUM2 and by weakening the binding of PUM2 to the 3′ untranslated region (3′-UTR) of HIF-1α mRNA [156]. On the other hand, it enhances HIF-1α protein stability by promoting the degradation of PHD1 and PHD3 [69]. In addition, LMP1 can increase HIF-1α promoter activity and further amplify this signaling output through a positive feedback loop in which HIF-1α reinforces its own promoter activity [156].

In NPC, its function is mainly concentrated in metastatic dissemination, metabolic adaptation, and radioresistance. Recent studies make it clear that this pathway is not merely a passive response to oxygen deprivation. Hypoxia can drive NPC cells to release exosomes. Early work showed that the hypoxic exosomes were loaded with MMP-13 which could enhance migration and invasiveness and induce microenvironment changes to promote NPC aggressiveness [157]. In parallel, exosomes containing miR-455, which is regulated by HIF-1α, can increase vascular permeability by targeting ZO-1, thereby creating a more permissive environment for metastasis [158]. Hypoxia can also induce metabolic reprogramming in NPC. Available evidence indicates that, following LMP1‑mediated upregulation of HIF-1α, NPC cells exhibit increased glucose uptake, lactate production, and LDH activity, together with upregulation of glycolytic regulators such as GLUT1 and PDK1 [64, 159]. These changes help tumor cells sustain their energetic and biosynthetic demands under conditions of limited oxygen supply. Given the central role of radiotherapy in NPC treatment, hypoxia-induced radioresistance is of particular clinical relevance. Clinical studies have associated HIF-1α and VEGF expression with poorer outcomes after radiotherapy, while hypoxia-related imaging has also been explored for treatment stratification and response assessment [160, 161]. More recent work has further shown that HIF-1α promotes radioresistance in NPC by transcriptionally upregulating hypoxia-inducible lipid droplet-associated protein (HILPDA), thereby enhancing lipid droplet formation and suppressing ferroptosis [162]. From a therapeutic perspective, a hypoxia-tropic nanozyme delivery strategy targeting transferrin receptor 1 enhanced radiosensitivity in preclinical NPC models [163]. Hypoxia also contributes to immunosuppression. HIF-1α upregulates exosomal PD-L1 expression in NPC, and macrophages can suppress CD8 + T cell activity through uptake of exosomal PD-L1 [164].

#### Integrated pathway crosstalk in NPC

Although these pathways are discussed separately, they should not be viewed as isolated signaling modules in NPC. EBV latent proteins, particularly LMP1, can simultaneously engage multiple downstream cascades, including NF-κB [35], PI3K/AKT [36], JAK/STAT3 [65] and MAPK signaling [125]. Therefore, these pathways may function as an interconnected network rather than as independent linear routes. LMP1-induced GLUT1 expression depends on coordinated mTORC1 and NF-κB signaling, linking viral oncogenic signaling with metabolic adaptation in NPC cells [64]. Crosstalk between NF-κB and STAT3 signaling has also been implicated in PD-L1 upregulation and immune escape within the NPC tumor vasculature [115]. In addition, LMP1 can coordinately activate STAT3, EGFR and ERK through PKCδ-dependent signaling, illustrating how a shared upstream regulator can integrate multiple oncogenic signaling outputs [65]. Divergent activation is also evident within the MAPK network. LMP1 can activate ERK/MAPK signaling through CTAR1-dependent mechanisms, whereas JNK/c-Jun activation is mediated through a TRAF6–TAK1/TAB1–JNKK1/2 cascade, indicating that the same viral oncoprotein can mobilize distinct MAPK modules through different upstream signaling routes [66, 133]. Functionally, these interconnected pathways converge on proliferation, survival, immune evasion and treatment resistance (Fig. 2).

This network-based view also has therapeutic implications. It may explain why inhibition of a single pathway can show strong preclinical activity but limited clinical translation, because compensatory signaling may maintain malignant phenotypes. Future therapeutic development should therefore prioritize biomarker-defined vulnerabilities and rational combinations, particularly those integrating pathway inhibition with radiotherapy, chemotherapy or immunotherapy.

### Genetic and epigenetic alterations

Compared with many solid tumors driven by recurrent canonical oncogenic mutations, NPC appears to be characterized more prominently by selective somatic lesions and extensive epigenetic dysregulation. Together, these abnormalities are thought to provide a molecular basis for persistent transcriptional dysregulation and the maintenance of malignant phenotypes [14, 15, 165] (Table 2).

**Table 2:** Genetic and epigenetic alterations in NPC

Alteration category	Gene(s)/target	Function	Ref	
Inactivating alteration	*ARID1A* *BAP1* *KMT2D* *KMT2C* *EP300*	Impairs chromatin remodeling and transcriptional control		[105, 166]
Inactivating alteration	*CYLD* *TRAF3* *NFKBIA*	Sustains NF-κB activation		[105]
Homozygous deletion	*CDKN2A/CDKN2B*	Disrupts cell-cycle restraint		[35]
Co-deletion	*MTAP*	Creates a potential MAT2A-dependent therapeutic vulnerability		[35]
Amplification	*CCND1* *CCND2* *CDK4* *CDK6*	Promotes cell-cycle progression		[35]
Amplification	*LTBR*	Activates NF-κB pathway		[35]
Somatic mutation	*TP53*	Disrupts p53-mediated tumor suppression		[35]
Homozygous deletion/structural alteration	*TGFBR2*	Promotes oncogenesis; stabilizes EBV infection		[35]
DNA methylation alterations	*RASSF1A*	Enables minimally invasive detection; acquisition of stem-like characteristics and invasive phenotypes		[167, 168]
	*BLU* *ADAMTS9* *DLEC1*	Silences tumor-suppressor		[169]
	*DERL3*	Promotes proliferation, migration, and invasion		[170]
	*SEPT9* *H4C6*	Enables minimally invasive detection		[167]
Chromatin regulatory	Histone modification	Represses the expression of DNA repair genes		[171]
	SOX2	Links super‑enhancer activity to chromatin reorganization		[172]
	*NOTCH3*	Promotes chemoresistance		[16]
	*ETV6*	Promotes oncogenic transcriptional programs		[173]
	*NDRG1* *TRIB1*	Promotes metastatic progression		[174]
	Inactive B compartments	Drives aberrant enhancer activation		[17]

#### Genomic alterations

Early integrative analyses of 128 NPC cases first highlight that NPC is characterized by a relatively low overall mutational burden and a dispersed distribution of altered genes, yet with clear enrichment of abnormalities affecting chromatin-remodeling pathways. In particular, tumor-suppressive chromatin regulators such as *ARID1A* and *BAP1* were found to undergo inactivating events, including mutation, deletion and loss of heterozygosity (LOH) [14]. Subsequent whole-exome sequencing (WES) and whole-genome sequencing (WGS) studies further reinforced the view. Recurrent alterations have been identified in negative regulatory factors such as *CYLD*, *TRAF3*, *NFKBIA* and *NLRC5,* while chromatin-modifying genes including *KMT2D, KMT2C* and *EP300* were also identified, although these events occurred at relatively low frequencies [105]. Collectively, these findings suggest that the genetic basis of NPC does not primarily depend on a small number of canonical oncogenes, but is more often shaped by the disruption of multiple tumor-suppressive network nodes. Genome-wide analyses further identified deletion of the 9p21.3 region as one of the most representative chromosomal events in NPC, which frequently involves homozygous deletion of the *CDKN2A/CDKN2B* locus and is often accompanied by nearby structural breakpoints [105]. In addition, co-deletion of the adjacent *MTAP* locus is commonly observed [105]. Fine mapping of deletion breakpoints involving *CDKN2A/CDKN2B* further supports this region as a critical genomic vulnerability in NPC [35]. Notably, the sensitivity of *MTAP*-deleted NPC cells to *MAT2A* inhibition also raises the possibility that *MTAP* loss may define a therapeutically actionable vulnerability [35]. More broadly, genome-wide remodeling of the cell-cycle regulatory network appears to be a common feature of NPC. In addition to *TP53* alterations, amplification of *CCND1*, *CCND2*, *CDK4* and *CDK6* is also prominent, indicating that cell-cycle dysregulation constitutes an important component of NPC genomic reprogramming and provides a key genetic basis for sustained proliferative capacity [35].

#### DNA methylation alterations

**Fig. 3:**
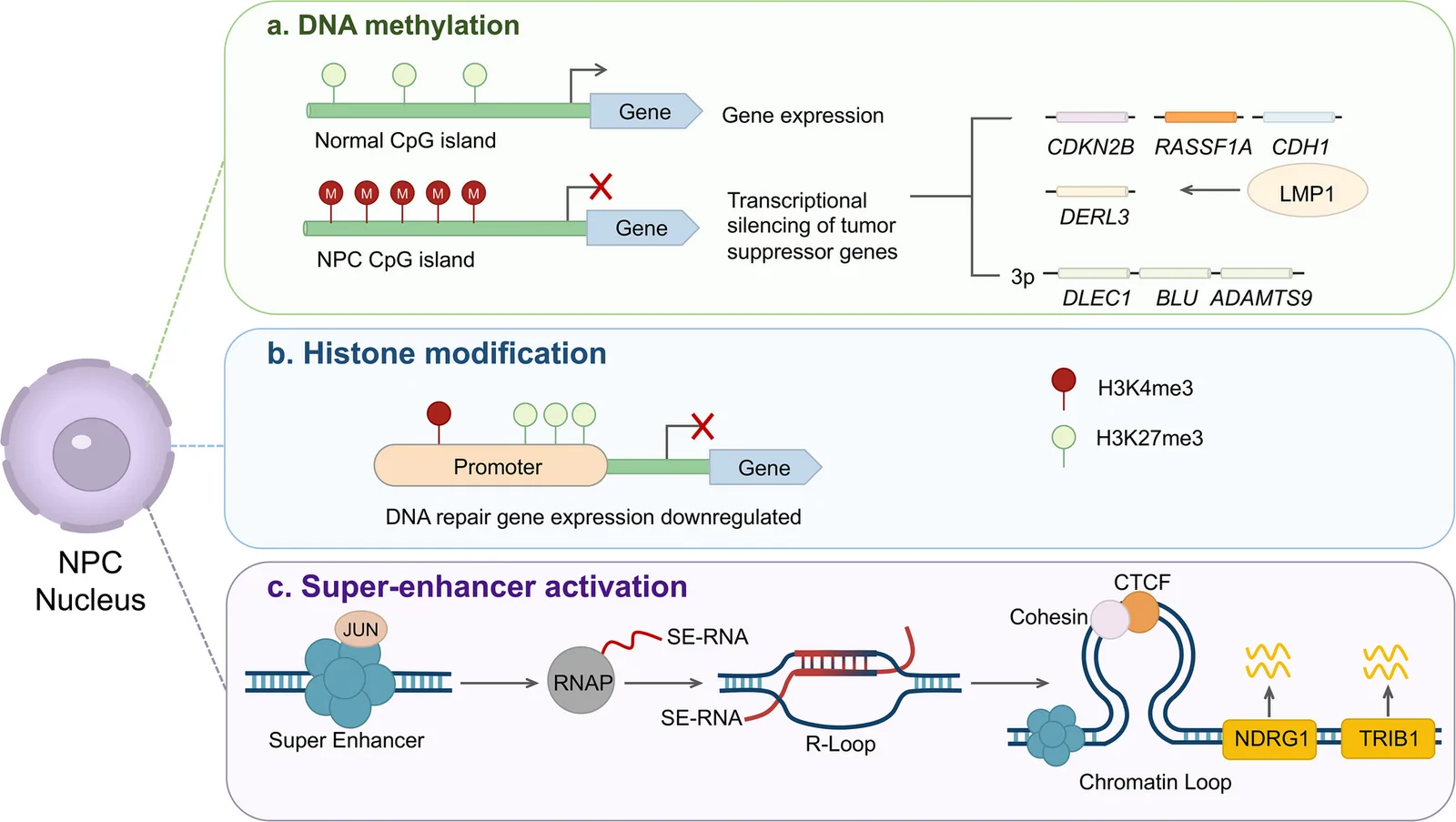
Epigenetic and chromatin regulatory alterations in NPC. **a** Aberrant promoter CpG island methylation contributes to transcriptional silencing of tumor-suppressor genes in NPC, including CDKN2B, RASSF1A, CDH1, DERL3, and tumor-suppressor genes located on chromosome 3p. LMP1 may further contribute to aberrant DNA methylation. **b** Altered histone modifications, characterized by reduced H3K4me3 and increased H3K27me3 at selected promoters, are associated with downregulation of DNA repair gene expression. **c** Aberrant super-enhancer activity and enhancer RNA-associated R-loop formation and chromatin looping promote oncogenic transcriptional programs, including activation of metastasis-related genes such as NDRG1 and TRIB1. Abbreviations: CTCF, CCCTC-binding factor; H3K4me3, trimethylation of histone H3 lysine 4; H3K27me3, trimethylation of histone H3 lysine 27; LMP1, latent membrane protein 1; NPC, nasopharyngeal carcinoma; RNAP, RNA polymerase; SE-RNA, super-enhancer RNA

DNA methylation is an important form of chemical DNA modification that can alter gene expression without changing the DNA sequence, and can influence chromatin structure, DNA conformation, genomic stability, and DNA–protein interactions [175, 176]. Compared to other tumors, NPC has a more pronounced hypermethylation profile, and a higher methylation burden has also been associated with poorer survival outcomes [177]. DNA methylation abnormalities are not restricted to individual genes, but are commonly characterized by widespread hypermethylation affecting promoter CpG islands and their adjacent regulatory regions [178]. Studies have shown that the methylation targets most consistently reported in NPC are mainly involved in cell-cycle inhibition, growth suppression, and maintenance of the epithelial phenotype [179]. Early candidate-gene studies identified differential methylation of multiple genes in undifferentiated NPC, with methylation frequencies of 50%, 50%, and 46% for *CDH1*, *CDKN2B*, and *RASSF1A* [180]. Among these, *RASSF1A* promoter hypermethylation was further confirmed as a frequent event in NPC [181]. More recent studies have suggested that methylation abnormalities are not randomly distributed, but instead tend to cluster in tumor-suppressor genes located within specific chromosomal loss regions. For example, the 3p tumor suppressors *BLU*, *ADAMTS9* and *DLEC1* all show high frequencies of promoter hypermethylation in NPC, with corresponding methylation rates of 80.65%, 62.37%, and 74.19% [169]. This suggests that tumor suppressor genes within chromosomal deletion regions are often subject to an additional layer of epigenetic transcriptional repression. LMP1 may be a key factor inducing aberrant DNA methylation of multiple tumor-suppressor genes in NPC. *DERL3* is the methylated gene most significantly affected by LMP1, whose low expression is associated with enhanced cell proliferation, migration, and invasion [170]. DNA methylation also has potential value in early cancer screening. Multi-gene methylation panels including *SEPT9, H4C6*, and *RASSF1A* have shown potential diagnostic and screening value, and detection of these methylation events in nasopharyngeal swabs may serve as a minimally invasive tool for NPC diagnosis [167] (Fig. 3a).

These observations indicate that DNA methylation in NPC is not merely a consequence of malignant transformation. Instead, it appears to participate actively in tumor-suppressor silencing and early tumor detection. The coexistence of promoter hypermethylation with chromosomal loss also suggests that genetic and epigenetic mechanisms may cooperate to disable tumor-suppressive programs. Therefore, methylation alterations may be particularly valuable when used as multi-marker panels rather than single-gene assays, but their clinical implementation will require validation across endemic and non-endemic populations.

#### Chromatin regulatory abnormalities

Chromatin regulatory dysregulation operates at multiple levels in NPC. It encompasses not only histone modification and dysfunction of chromatin remodeling complexes, but also alterations in enhancers, super-enhancers and higher-level chromatin architecture [35, 172]. Histone modifications represent a fundamental layer of chromatin regulation [182]. Studies have shown that, in EBV-associated nasopharyngeal epithelial cells, the promoters of certain DNA repair genes display reduced H3K4me3 and increased H3K27me3, accompanied by downregulation of the corresponding transcripts [171]. Aberrations in chromatin remodeling complexes likewise constitute an important component of chromatin dysregulation in NPC, and these complexes serve as core executors of nucleosome positioning, chromatin accessibility and transcriptional competence. Whole-genome and exome sequencing studies have identified somatic alterations in multiple chromatin regulatory genes, and mutations or deletions affecting these factors provide an important genetic basis for chromatin disruption in NPC by impairing complex assembly or activity and thereby perturbing transcriptional control [35, 105, 166] (Fig. 3b).

Abnormalities in histone modification and chromatin remodeling complexes primarily lead to the repression of expression in some genes. At a higher level of chromatin regulation, enhancers and super‑enhancers are often abnormally activated, thereby promoting the overexpression of tumor‑related genes. Super-enhancers are large clusters of constituent enhancers with exceptionally high regulatory activity, and they typically control genes that are critical for cell identity or tumor maintenance. Early H3K27ac ChIP-seq profiling showed that NPC harbors a set of tumor-specific enhancers and super-enhancers that are newly activated [173]. These regions are enriched for binding motifs of transcription factors such as NF-κB, IRF1/2 and ETS1, and are linked to oncogenesis-related genes, including ETV6 [173]. Subsequent studies further delineated an aberrant super-enhancer landscape in NPC and identified SOX2 as a super-enhancer ‑driven oncogenic dependency [172]. Importantly, the SOX2-associated super-enhancer does not merely accompany elevated SOX2 expression, rather, it participates in local chromatin reorganization, promotes enhancer–promoter interaction, and contributes directly to tumor growth [172]. These findings suggest that the significance of super-enhancer dysregulation in NPC lies not simply in increased enhancer activity, but in the establishment of new transcriptional dependencies that help sustain malignant growth.

Enhancer reprogramming has also been linked to treatment resistance. In advanced NPC, the acquisition of chemoresistance is accompanied by genome-wide enhancer reprogramming, which drives aberrant NOTCH3 activation. Activated NOTCH3, in turn, promotes chemoresistance-associated phenotypes through the NOTCH3–SLUG axis, whereas genetic or pharmacological inhibition of NOTCH3 can at least partially restore sensitivity to paclitaxel [16]. More recently, this field has extended into the three-dimensional genome. One study showed that a JUN-dependent super-enhancer RNA can form R-loops within super-enhancer regions, thereby facilitating chromatin looping and long-range interaction between the super-enhancer and its target promoter. Through this mechanism, expression of the metastasis-associated gene *NDRG1* and its neighboring gene *TRIB1* is coordinately upregulated, promoting metastatic progression in NPC [174]. This finding indicates that chromatin dysregulation in NPC is not confined to enhancer activation, but may also involve enhancer RNAs, R-loop formation and topological chromatin remodeling acting in concert (Fig. 3c).

In EBV-positive NPC, the viral genome can also directly participate in this chromatin reorganization. EBV episomes have been shown to interact preferentially with inactive B compartments of the host genome, and these EBV-interacting regions display increased levels of active histone marks in NPC cells [17]. This phenomenon has been described as “enhancer infestation”, whereby EBV reshapes host chromatin through virus–host interaction and induces aberrant enhancer activation in regions that would otherwise remain relatively silent [17]. Together, these observations indicate that chromatin dysregulation in NPC is not solely the consequence of host-cell intrinsic regulatory imbalance, but also reflects a close interplay between viral persistence and host chromatin architecture.

## Clinical features

NPC most commonly arises in the pharyngeal recess [183]. Owing to the deep anatomical location of the nasopharynx, early lesions are often not readily detectable, which makes early diagnosis challenging [1, 184]. Clinically, most NPC patients are diagnosed with locoregionally advanced disease, and approximately 10% already have distant metastases at initial presentation [1]. Early symptoms are usually subtle and nonspecific, including nasal obstruction or minor blood-stained nasal discharge, and can therefore be easily mistaken for benign conditions [1, 184]. As the tumor extends toward the pharyngeal opening of the Eustachian tube and the surrounding structures, patients may develop otologic symptoms, including tinnitus, hearing impairment, ear fullness, or otitis media with effusion [185]. Therefore, the presence of unilateral ear symptoms in adult patients is often a telling sign, and increased vigilance should be exercised regarding NPC.

The nasopharynx is richly supplied by lymphatic networks, which underlies the high frequency of cervical lymph node metastasis in NPC, most commonly involving the retropharyngeal and level II neck nodes [186]. Painless cervical lymphadenopathy is one of the most common presenting features reported in NPC [187]. Therefore, NPC should be included in the differential diagnosis of unexplained cervical lymph node enlargement, especially when it is accompanied by sinonasal or otologic symptoms such as nasal obstruction, epistaxis, tinnitus, or hearing loss. Once the tumor extends beyond the mucosa into the parapharyngeal space, skull base, or adjacent neural pathways, headache often develops and may progressively worsen. The occurrence of diplopia, facial numbness, or other cranial nerve deficits usually suggests skull base extension or involvement of the relevant cranial nerves [188]. Cranial nerve involvement is generally regarded as a feature of advanced local disease and has been associated with an unfavorable prognosis [189]. In advanced-stage disease, distant dissemination may also occur, with bone, lung, and liver representing the most common metastatic sites; among these, bone metastasis is the most frequent [190]. These clinical features explain why NPC is frequently diagnosed only after nodal disease or skull-base-related symptoms have emerged. Therefore, heightened clinical suspicion, targeted evaluation of high-risk individuals and timely use of nasopharyngoscopy and imaging remain essential for reducing diagnostic delay in endemic regions.

In addition, pediatric NPC represents a distinct clinical setting that differs from adult disease in presentation, treatment tolerance and survivorship considerations. It is predominantly non-keratinizing or undifferentiated histologically and often presents as locally advanced [191]. Nevertheless, pediatric NPC generally responds well to chemotherapy combined with radiotherapy and has a good long-term prognosis [192]. Therefore, managing late toxicities and survival care is particularly important.

## Diagnosis and staging

The deep anatomical location of the nasopharynx, together with the predominantly submucosal growth pattern of early lesions, often makes timely detection difficult on the basis of symptoms and physical examination. Therefore, patients suspected of having NPC should undergo a comprehensive head and neck examination. Endoscopy-guided biopsy of the primary nasopharyngeal lesion remains the definitive method for establishing the diagnosis [184]. However, submucosal or very small lesions may be occult on endoscopic examination. MRI can identify subclinical lesions missed by endoscopy and endoscopic biopsy and therefore provides an important complementary approach for early detection [18, 193]. In population-based screening settings, MRI has also shown higher sensitivity than endoscopy, particularly for early-stage disease [194]. Accordingly, targeted biopsy should be considered when MRI or positron emission tomography (PET) reveals a suspicious nasopharyngeal lesion despite inconclusive endoscopic findings [184].

EBV-related biomarkers provide an additional approach for identifying individuals who warrant further diagnostic evaluation. Among them, plasma EBV DNA has substantial clinical evidence for screening in endemic populations. In a large prospective study involving 20,174 asymptomatic participants, repeated plasma EBV DNA testing followed by endoscopy and MRI in individuals with persistently positive results achieved a sensitivity of 97.1% and a specificity of 98.6%; notably, 71% of screen-detected NPCs were diagnosed at stage I or II, compared with 20% in a historical cohort [45]. Subsequent expert recommendations have supported EBV-based screening of middle-aged adults in high-risk regions and individuals with a family history of NPC in intermediate-risk regions, with endoscopy and MRI used to investigate screen-positive individuals [195]. Nevertheless, plasma EBV DNA should not be used as a stand-alone test to exclude NPC, because a subset of patients, particularly those with low-volume or early-stage disease, may have undetectable plasma EBV DNA [196].

NPC is currently staged according to the ninth version of the American Joint Committee on Cancer/Union for International Cancer Control (AJCC/UICC) tumor–node–metastasis (TNM) classification [197]. Compared with TNM-8, TNM-9 incorporates advanced radiological extranodal extension into the N3 category, regroups nonmetastatic disease into stages I–III, and confines stage IV to metastatic disease, with further subdivision according to metastatic burden [197]. Imaging remains central to accurate anatomical staging. MRI is the preferred modality for assessing the primary tumor and regional nodal involvement because of its superior soft-tissue resolution, whereas ^18F-fluorodeoxyglucose (^18F-FDG) PET/CT provides complementary value for detecting distant metastases, particularly in patients at higher metastatic risk [19, 184]. Although TNM-9 improves anatomical prognostic stratification, it remains fundamentally anatomy based, and its development provides a framework for future incorporation of nonanatomical prognostic factors, particularly plasma EBV DNA [197]. Pretreatment plasma EBV DNA has established prognostic value in EBV-associated NPC, and studies based on TNM-8 have shown that incorporating plasma EBV DNA into conventional TNM-based models can improve prognostic discrimination [198, 199]. However, plasma EBV DNA has not been incorporated as a formal component of TNM-9 [197]. One major obstacle to broader clinical implementation is assay standardization. PCR-based EBV DNA assays remain heterogeneous across laboratories, and harmonized and validated assays are required for reliable cross-center comparison [200]. Although international harmonization studies have shown that standardized assay conditions and common calibrators can improve interlaboratory agreement, universally validated thresholds for different clinical applications remain lacking [201]. Therefore, plasma EBV DNA is currently better regarded as a complementary biological risk marker for refining prognostic stratification rather than as a formal component of anatomical staging. Serial plasma EBV DNA measurements during and after treatment can also aid response assessment and recurrence surveillance [202]. Beyond EBV DNA, other factors, including primary-tumor gross tumor volume (GTV), nodal GTV, neutrophil-to-lymphocyte ratio (NLR), lactate dehydrogenase (LDH), C-reactive protein/albumin ratio, platelet count, SUVmax, and total lesion glycolysis, may also contribute to further refinement of the current staging framework [203].

## Management and treatment

**Fig. 4:**
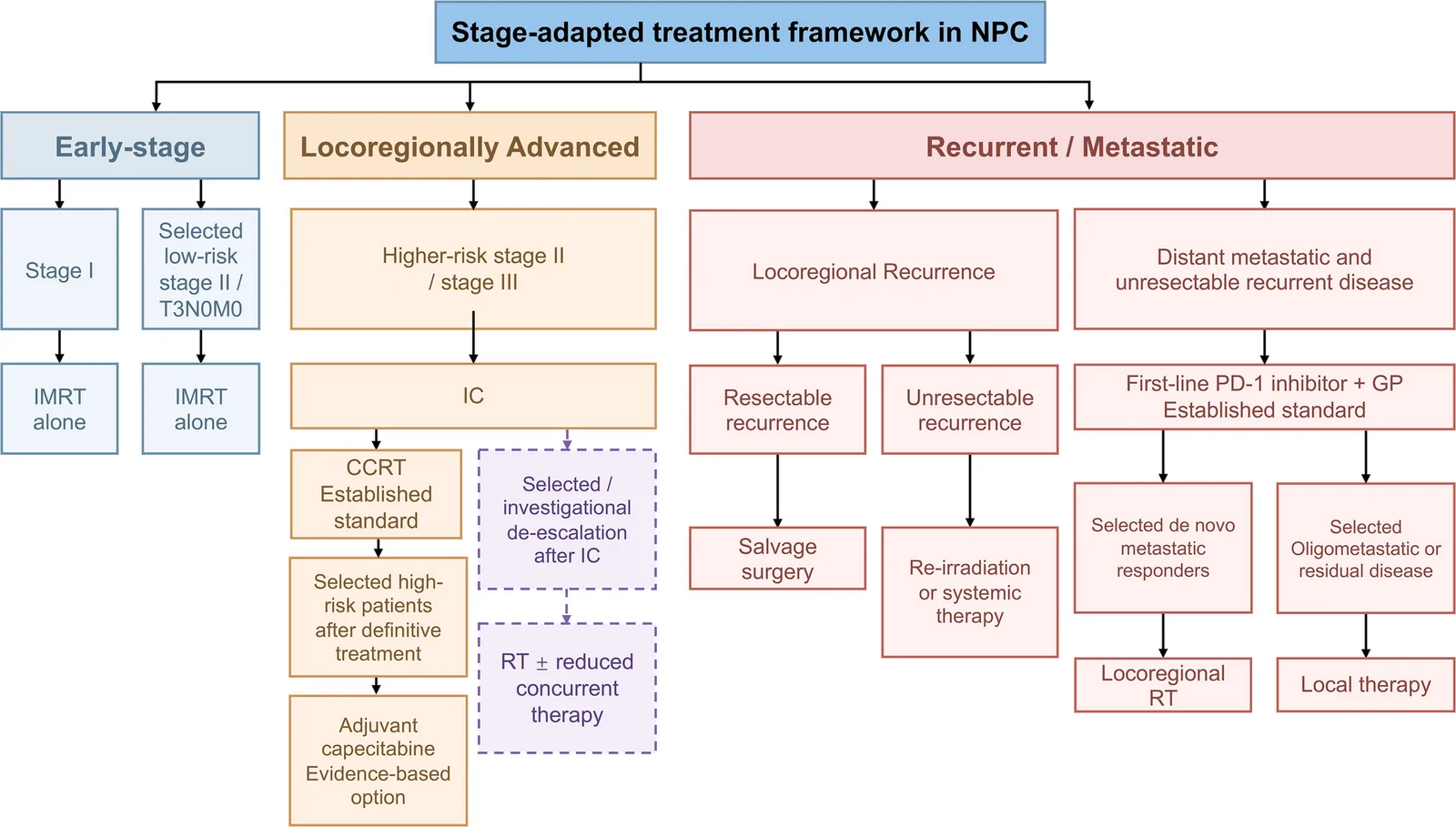
Stage-adapted treatment framework in NPC. Treatment of nasopharyngeal carcinoma is adapted according to disease stage, recurrence pattern, resectability, metastatic burden, and treatment response. Stage I and selected low-risk stage II/T3N0M0 disease may be managed with intensity-modulated radiotherapy alone. For higher-risk stage II and stage III disease, induction chemotherapy followed by concurrent chemoradiotherapy represents an established treatment approach, whereas selected de-escalation strategies after induction chemotherapy, including radiotherapy with or without reduced concurrent therapy, remain investigational. Adjuvant capecitabine represents an evidence-based option for selected high-risk patients after definitive treatment. For locoregional recurrence, salvage surgery or re-irradiation/systemic therapy may be considered according to resectability. For metastatic or unresectable recurrent disease, first-line PD-1 inhibitor plus gemcitabine–cisplatin represents an established systemic treatment. Locoregional radiotherapy may be considered in selected patients with de novo metastatic disease who respond to systemic therapy, whereas local therapy may be used in selected patients with oligometastatic or residual disease. Abbreviations: CCRT, concurrent chemoradiotherapy; GP, gemcitabine plus cisplatin; IC, induction chemotherapy; IMRT, intensity-modulated radiotherapy; PD-1, programmed cell death protein 1; RT, radiotherapy

Treatment is based on staging, recurrence risk, and prior treatment history in NPC [184, 204] (Fig. 4). Unlike other head and neck tumors, NPC treatment is not surgery‑based, but relies on radiotherapy and its combination with systemic therapy [184]. For early-stage cases, the focus is on achieving stable local control through radical radiotherapy, while for locally advanced patients, chemotherapy is usually combined with radiotherapy to control distant metastases and prolong long-term survival [205, 206]. Once the disease progresses to recurrence or metastasis, the focus shifts from local radical treatment to systemic disease control, with local therapy serving more as a supplementary measure in specific situations [207]. In addition, as survival outcomes have improved, treatment decisions in NPC are increasingly required to balance disease control with long-term toxicity and quality of life. Because radiotherapy is central to NPC treatment, long-term toxicities such as xerostomia, hearing impairment, otitis media, dysphagia, neck fibrosis, cranial neuropathy, endocrine dysfunction and dental complications can substantially affect survivorship [208]. These toxicities are closely related to the anatomical location of the nasopharynx. Treatment optimization should therefore consider not only tumor control and survival, but also long-term quality of life [209]. Current treatment standards differ according to disease setting. IMRT remains the radiotherapy backbone for nonmetastatic NPC, while cisplatin-based concurrent chemoradiotherapy, often preceded by induction chemotherapy in patients with locoregionally advanced disease, represents an established treatment approach [19, 206]. For recurrent or metastatic NPC not amenable to curative local treatment, first-line PD-1 blockade combined with gemcitabine–cisplatin has become an established treatment option, as demonstrated in several phase III trials and reflected in current clinical recommendations [22, 204, 210, 211]. By contrast, omission or redistribution of concurrent chemotherapy after induction chemotherapy and the incorporation of PD-1 blockade into curative-intent chemoradiotherapy remain evolving strategies that require further validation before routine adoption [212–215].

### Management of early-stage NPC

Treatment of early-stage NPC is based on radical radiotherapy, with the core objective of minimizing long-term toxicity while ensuring local control [205, 216]. Among current techniques, intensity-modulated radiotherapy (IMRT) remains the preferred radiotherapy technique and is an established standard for T1N0M0 disease [19, 184]. For patients with other early-stage disease, the role of concurrent chemotherapy is less uniform and should be considered in the context of additional risk factors. Contemporary recommendations support radiotherapy alone for many patients with early-stage disease, while concurrent chemotherapy may be considered in those with higher-risk features such as N1 disease, bulky T2 tumors, or elevated pretreatment EBV DNA [207]. This risk-adapted approach is supported by randomized phase III evidence. In a multicenter noninferiority trial of 341 patients with low-risk stage II/T3N0M0 NPC, defined by the absence of adverse nodal features and a pretreatment EBV DNA level below 4000 copies/mL, IMRT alone was noninferior to concurrent chemoradiotherapy in terms of 3-year failure-free survival (90.5% vs 91.9%) and was associated with substantially fewer grade 3–4 adverse events (17% vs 46%) [217]. A subsequent meta-analysis likewise found no clear survival advantage from adding concurrent chemotherapy to IMRT in stage II or T3N0M0 disease, while treatment-related toxicity was increased [218]. These findings support omission of concurrent chemotherapy as an evidence-based de-escalation option for carefully selected low-risk patients rather than as a routine strategy for all patients with early-stage NPC.

### Management of locoregionally advanced NPC

Cisplatin-based concurrent chemoradiotherapy (CCRT) remains an established treatment backbone for locoregionally advanced NPC, while induction chemotherapy followed by CCRT (IC + CCRT) is widely used for patients at higher risk of distant failure [206]. Randomized phase III trials have established the benefit of adding induction chemotherapy to CCRT. Gemcitabine–cisplatin (GP) induction chemotherapy followed by CCRT improved overall survival compared with CCRT alone, with no apparent increase in late toxicities [20]. Long-term follow-up of a phase III trial of TPF induction chemotherapy likewise demonstrated significant improvements in failure-free survival, overall survival, and distant disease control [219]. The updated MAC-NPC individual-patient-data network meta-analysis further showed that adding induction or adjuvant chemotherapy to CCRT improved overall survival compared with CCRT alone [220].

However, as IC has been increasingly incorporated upfront, whether full-intensity concurrent cisplatin remains necessary after induction chemotherapy has come under renewed scrutiny. A randomized trial showed that, in patients with locoregionally advanced NPC who had received induction chemotherapy, subsequent radiotherapy alone was noninferior to CCRT in terms of 3-year progression-free survival and was associated with lower acute toxicity [212]. These findings suggest that treatment intensity may be reduced in selected patients after induction chemotherapy. Another randomized trial further evaluated a sequential chemoradiotherapy approach, consisting of induction chemotherapy followed by radiotherapy and then adjuvant GP. The results indicated that this strategy achieved efficacy comparable to that of induction chemotherapy plus CCRT while reducing acute toxic effects [213]. Nevertheless, these approaches should currently be viewed as risk-adapted de-escalation strategies rather than routine replacements for IC + CCRT.

For patients who remain at high risk of recurrence after definitive chemoradiotherapy, how to deliver subsequent adjuvant treatment without substantially increasing toxicity has also become an important focus of recent research. In this context, oral adjuvant strategies that can be delivered over a prolonged period have attracted growing attention. In a randomized clinical trial, full-dose adjuvant capecitabine improved failure-free survival in patients with high-risk locoregionally advanced NPC after CCRT and was generally well tolerated [21]. Notably, oral capecitabine is not limited to the conventional full-dose schedule in the adjuvant setting. In recent years, increasing attention has been directed toward metronomic chemotherapy, which refers to the frequent and regular administration of oral chemotherapeutic agents at relatively low doses over a prolonged period [221]. A phase III trial demonstrated that, in high-risk locoregionally advanced NPC, metronomic capecitabine administered after definitive chemoradiotherapy significantly improved failure-free survival, with a manageable safety profile [222]. These findings support capecitabine-based adjuvant therapy as an evidence-based option for selected high-risk patients, although it should not be interpreted as a universal requirement after definitive treatment.

Plasma EBV DNA may further help identify patients who remain at increased risk during and after definitive treatment. In a prospective study, longitudinal plasma cell-free EBV DNA measurements during treatment were closely associated with dynamic changes in recurrence risk, supporting their potential use for treatment-response assessment and risk stratification [223]. However, whether EBV DNA-defined risk should directly determine treatment intensification remains uncertain. In a randomized trial, patients with detectable plasma EBV DNA 6–8 weeks after radiotherapy were identified as a high-risk group, but adjuvant cisplatin plus gemcitabine did not improve relapse-free survival compared with observation [224]. Therefore, serial plasma EBV DNA is currently more useful for response assessment and risk stratification than as a stand-alone criterion for selecting additional treatment.

PD-1 blockade is also being incorporated into curative-intent treatment for high-risk locoregionally advanced NPC. In a phase III trial, adding sintilimab to induction-concurrent chemoradiotherapy improved 3-year event-free survival from 76% to 86%, although grade 3–4 adverse events were more frequent with sintilimab [214]. More recently, a phase III trial in patients who remained at high risk after GP induction chemotherapy showed that concurrent and adjuvant camrelizumab added to CCRT improved 3-year progression-free survival [215]. These phase III data support the potential integration of PD-1 blockade into definitive treatment for selected high-risk patients. However, unlike first-line chemoimmunotherapy for recurrent or metastatic NPC, the optimal patient selection, sequencing, and duration of immunotherapy in the curative setting remain to be defined, and these regimens are not yet uniformly established as routine treatment across clinical settings.

### Management of recurrent or metastatic NPC

Recurrent or metastatic NPC (R/M NPC) is a clinically heterogeneous entity, and treatment should be individualized according to the pattern of recurrence or dissemination, previous treatment, resectability, metastatic burden, and performance status. Locoregionally recurrent disease may still be amenable to curative-intent local treatment in selected patients, whereas systemic therapy becomes the main treatment for distant metastatic disease or recurrence not suitable for salvage local therapy [207]. For technically resectable local or regional recurrence, salvage surgery may be considered when feasible, while re-irradiation remains an important option for patients who are not suitable surgical candidates [207]. In patients requiring re-irradiation for locally advanced recurrent NPC, a randomized phase III trial showed that hyperfractionated IMRT reduced severe late toxicity and improved overall survival compared with standard fractionation [225].

For patients with metastatic disease or unresectable recurrence requiring systemic therapy, the first-line standard has shifted from GP chemotherapy alone to PD-1 blockade combined with GP [207]. This change is supported by consistent phase III evidence. Toripalimab plus GP significantly prolonged both progression-free and overall survival compared with GP alone [22]. Subsequent study further showed that the addition of camrelizumab to GP not only delayed disease progression, but also conferred a sustained overall survival benefit, reducing the risk of death by 26% and increasing the absolute 5-year survival rate by 13.6%, suggesting that approximately one-third of patients may achieve a relatively durable survival plateau [226]. Long-term follow-up demonstrated sustained progression-free survival benefit and improved overall survival with tislelizumab plus GP [227]. Collectively, these trials establish PD-1 inhibitor plus GP as a first-line standard rather than an investigational strategy for R/M NPC, although the availability and regulatory status of individual PD-1 inhibitors vary across regions [207]. In contrast, no universally established second-line standard exists after progression on contemporary first-line chemoimmunotherapy, and subsequent systemic treatment remains individualized.

Local therapy may also have a role in selected metastatic settings. In a randomized phase III trial of patients with de novo metastatic NPC who achieved a complete or partial response to initial chemotherapy, the addition of locoregional radiotherapy significantly improved overall survival compared with chemotherapy alone [228]. Therefore, locoregional radiotherapy is an evidence-based option for carefully selected, chemotherapy-sensitive patients with de novo metastatic disease rather than a routine approach for all metastatic NPC. Local treatment of metastatic sites may be considered in selected patients with oligometastatic disease [207, 229].

### Emerging and investigational therapeutic strategies

Although radiotherapy, chemotherapy, and PD-1 inhibitors have substantially improved the treatment landscape of NPC, important limitations remain for patients with refractory recurrence, distant metastasis, or treatment-resistant disease. Therefore, multiple therapeutic strategies continue to be actively explored. In recent years, therapeutic research in NPC has gradually shifted from conventional radiotherapy and chemotherapy toward precision approaches guided by molecular features, virus-associated targets, and the tumor microenvironment. The clinical maturity of these strategies varies considerably, and most remain investigational, but some have begun to enter clinical practice in defined patient populations.

Antibody–drug conjugates (ADCs) are among the most clinically advanced targeted strategies in NPC. By combining antigen-specific antibodies with cytotoxic payloads, ADCs enable selective delivery of potent anticancer agents to tumor cells [230]. In NPC, the most actively investigated targets currently include EGFR and B7-H3. Becotatug vedotin (MRG003) is an EGFR-targeted ADC that has progressed furthest in clinical development. In a randomized multicenter study of heavily pretreated R/M NPC after failure of at least two lines of systemic therapy, including PD-1/PD-L1 inhibitors, becotatug vedotin achieved a higher objective response rate than investigator-selected chemotherapy and significantly prolonged progression-free survival, while overall survival data remained immature [231]. These findings supported its regulatory approval for adult patients with R/M NPC who had failed at least two lines of systemic chemotherapy and PD-1/PD-L1 inhibitor therapy in China [232]. Thus, becotatug vedotin is no longer purely investigational in this specific treatment setting, although its role outside the approved indication remains under evaluation. A randomized phase III study is currently assessing becotatug vedotin combined with pucotenlimab versus chemotherapy in previously treated R/M NPC (NCT06976190). B7-H3 represents another emerging ADC target in NPC. YL201, a B7-H3-targeted ADC, showed encouraging activity in a phase I/Ib study of advanced solid tumors, with an objective response rate of 48.6% among patients with NPC [233]. A randomized phase III trial is now comparing YL201 with investigator-selected chemotherapy in patients with R/M NPC who have failed prior PD-L1 inhibitor therapy and at least two lines of chemotherapy (NCT06629597). YL201 therefore remains investigational, although its development has advanced into late-phase clinical evaluation.

Antiangiogenic therapy represents another clinically relevant area of investigation, particularly in later-line treatment and in combination with immune checkpoint blockade. In a single-arm phase II study, camrelizumab plus the VEGFR-2 inhibitor apatinib achieved an objective response rate of 65.5% after failure of at least one prior line of systemic therapy, although grade ≥ 3 treatment-related adverse events were frequent [234]. Activity was also observed in platinum-resistant and PD-1 inhibitor-resistant disease in a subsequent phase II study [235]. Anlotinib has likewise shown antitumor activity both as later-line monotherapy and in combination with toripalimab in single-arm phase II trials [236, 237]. Overall, these findings support further evaluation of antiangiogenic strategies, particularly in combination with PD-1 blockade. However, the available evidence remains largely limited to single-arm phase II studies, and randomized confirmatory data are lacking; these regimens therefore remain investigational rather than established later-line treatment standards.

EBV-directed immunotherapy is an important investigational direction that distinguishes NPC from many other head and neck cancers. Because non-keratinizing NPC is closely associated with EBV infection, EBV latent antigens can provide relatively tumor-selective immune targets [5]. Among these approaches, EBV-specific cytotoxic T lymphocytes (CTLs) were among the earliest therapeutic strategies to enter clinical investigation. These cells can recognize and eliminate EBV-positive tumor cells with antigen-directed specificity [238]. Early studies demonstrated that autologous EBV-specific CTLs could be safely administered to patients with advanced or recurrent NPC and were associated with durable responses in a subset of patients [239, 240]. Attempts to improve their activity through preparative lymphodepletion, however, did not enhance clinical benefit [241]. More importantly, this early activity was not confirmed in randomized phase III testing. In a phase III trial of 330 patients with R/M NPC, gemcitabine–carboplatin followed by autologous EBV-specific CTLs did not improve overall survival compared with chemotherapy alone [242]. Thus, EBV-specific CTL therapy remains investigational despite earlier evidence of clinical activity. Genetically engineered cellular therapies, including EBV-directed CAR-T and TCR-T cells, are at an even earlier stage of development [243]. An ongoing early phase I study is evaluating their safety, dose tolerance, and preliminary activity in recurrent or refractory EBV-positive NPC (NCT05587543). Therapeutic EBV vaccines are designed to induce antigen-specific immune responses against EBV-positive tumor cells by delivering viral tumor antigens or their coding sequences [244, 245]. Their clinical development remains at an early stage. In phase I studies, the MVA-EL vaccine encoding an EBNA1/LMP2 fusion antigen showed acceptable safety and induced or enhanced EBV-specific T-cell responses in patients with NPC [244]. However, this study primarily assessed safety and immunogenicity, and therapeutic efficacy has not been established. More recently, an LMP2-mRNA lipid-delivery vaccine has shown antitumor potential in preclinical models of EBV-associated tumors [246]. Another study showed that LMP2-mRNA lipid nanoparticles can sensitize EBV-associated tumor models to PD-1 blockade, suggesting that combining EBV-mRNA vaccines with immune checkpoint inhibitors warrants further investigation [247]. These findings provide a rationale for further clinical evaluation, but EBV-targeted therapeutic vaccines remain investigational.

Beyond these strategies, other targeted approaches are also being explored, mainly involving DNA damage repair, apoptosis regulation, and epigenetic abnormalities (Table 3). However, most of these approaches remain in preclinical or early-phase development and are still some distance from routine clinical application. Overall, NPC treatment is gradually moving beyond conventional radiotherapy and chemotherapy toward more precise therapeutic models based on molecular features, virus-associated targets, and the tumor microenvironment. Whether these strategies can be incorporated into routine treatment systems will require further validation in clinical studies.

**Table 3:** Emerging and investigational therapeutic strategies in NPC

Therapeutic class	Candidate agent(s)	Target	Development stage	Clinical trial identifier(s)/Ref
Antibody–drug conjugate (ADC)	Becotatug vedotin (MRG003)	EGFR	Approved in China for selected heavily pretreated R/M NPC; ongoing phase III	[232]; NCT05126719; NCT06976190
	SYS6010/CPO301	EGFR	Ongoing Phase I	ChiCTR2300072141
	izalontamab brengitecan (BL-B01D1)	EGFR/HER3	Ongoing phase III	NCT06118333
	YL201	B7-H3	Ongoing phase III	NCT06629597
Antiangiogenic therapy	Camrelizumab + apatinib	PD-1 + VEGFR-2	Completed phase II	NCT04586088
	Anlotinib	VEGFR/FGFR/PDGFR/c-Kit	Completed phase II	NCT03906058
	Toripalimab + anlotinib	PD-1 + multitargeted TKI	Completed phase II	NCT04996758
EBV-directed cellular therapy	EBV-specific CTLs	EBV latent antigens	Completed phase III	NCT02578641
	LMP1/LMP2-specific CTLs	LMP1/LMP2	Completed Phase I	NCT00516087
Engineered cellular therapy	EBV CAR-T/TCR-T	EBV-associated antigens	Ongoing phase I	NCT05587543
Therapeutic EBV vaccine	MVA-EBNA1/LMP2	EBNA1/LMP2	Completed Phase Ib; Completed phase II	NCT01800071; NCT01094405
	LMP2-mRNA vaccine	LMP2	Preclinical	[246]
	LMP2-mRNA lipid nanoparticle	LMP2	Preclinical	[247]
DNA repair targeting	Olaparib + pembrolizumab	PARP1 + PD-1	Ongoing phase II	NCT04825990
EBV lytic activation targeting	Valganciclovir Hydrochloride	EBV lytic induction	Ongoing phase II	NCT07138989
Apoptosis-targeted therapy	APG-1252 + gemcitabine	BCL-2/BCL-XL	Preclinical	[248]
Epigenetic therapy	JQ1	BET bromodomain proteins/c-Myc	Preclinical	[249]
	GdCPP	Imaging-guided tumor therapy	Preclinical	[250]
Nanomedicine-based therapy	Cancer-cell-membrane-shielded biomimetic nanoparticle	Homotypic tumor targeting	Preclinical	[251]
	Optical imaging/combined phototherapy nanoplatform	Photothermal/photodynamic therapy	Preclinical	[252]

Data sources: Published studies cited in the table; ClinicalTrials.gov and the Chinese Clinical Trial Registry (ChiCTR) for trial identifiers and development status

*Abbreviations*: *ADC* antibody–drug conjugate, *CTL* cytotoxic T lymphocyte, *EBV* Epstein–Barr virus, *EGFR* epidermal growth factor receptor, *FGFR* fibroblast growth factor receptor, *GP* gemcitabine plus cisplatin, *HER3* human epidermal growth factor receptor 3, *ICI* immune checkpoint inhibitor, *NPC* nasopharyngeal carcinoma, *PARP* poly(ADP-ribose) polymerase, *PD-1* programmed cell death protein 1, *PDGFR* platelet-derived growth factor receptor, *R/M* recurrent or metastatic, *TCR-T* T-cell receptor-engineered T cell, *TKI* tyrosine kinase inhibitor, *VEGFR* vascular endothelial growth factor receptor

## Conclusion and future perspectives

NPC is a distinctive malignancy in which viral infection, host susceptibility, and environmental exposure converge to shape disease initiation and progression. Unlike many other epithelial tumors, NPC is marked not only by its close association with EBV, but also by its characteristic geographic clustering, selective molecular alterations, extensive epigenetic dysregulation, and strong dependence on radiotherapy-based treatment. Over the past decades, progress in genomics, epigenomics, imaging, and systemic therapy has substantially expanded the current understanding of NPC. These advances increasingly support an integrated framework in which endemic distribution, EBV-driven oncogenesis, host-cell signaling dysregulation, and therapeutic evolution are linked rather than viewed as separate layers of disease.

Several challenges remain unresolved. Although plasma EBV DNA has proven clinically useful, its widespread use for routine risk stratification and staging is still constrained by assay variability, poor cross‑platform standardization, and limited detectability in some patients with low-volume or early-stage disease [45, 196, 200]. Similarly, the current TNM system remains indispensable for treatment decision-making, but it does not fully capture the biological heterogeneity of NPC or consistently predict prognosis and treatment benefit [198, 203]. At the molecular level, many signaling abnormalities and epigenetic events have been described, yet their relative hierarchy, context dependence, and therapeutic tractability remain incompletely defined. In particular, how EBV-derived signals interact with host genomic lesions, chromatin remodeling, immune escape, and treatment resistance still requires further clarification.

Therapeutic progress has improved outcomes, but treatment selection remains insufficiently precise. IMRT, induction chemotherapy, adjuvant capecitabine, and PD-1 inhibitor-based chemoimmunotherapy have improved outcomes in selected settings, yet locoregional recurrence, distant metastasis, radioresistance, and interpatient variability continue to limit long-term disease control [19, 20, 204, 222, 253]. These issues indicate that future progress in NPC management will depend not only on intensifying treatment, but also on improving treatment selection. More precise stratification is needed to identify which patients can safely undergo treatment de-escalation, which require intensified systemic therapy, and which are most likely to benefit from immunotherapy or other emerging strategies. In parallel, the toxicities associated with re-irradiation, combination treatment, and prolonged systemic therapy remain important clinical constraints that should be weighed more carefully in future studies.

Future research should focus on standardizing and validating clinically significant biomarkers, particularly plasma EBV DNA and other molecular or imaging-based biomarkers, to more reliably incorporate them into screening, staging, and efficacy assessment. Treatment refinement should move toward biologically informed risk stratification, allowing de-escalation in favorable-risk disease while reserving intensified systemic or investigational approaches for high-risk or treatment-resistant tumors. Additionally, research on targeted therapy may help move NPC management toward a more precise and individualized model. A deeper understanding of the interplay between viral oncogenesis, host molecular dysregulation, and clinical evolution will be essential for translating theory into durable benefit for patients with NPC.

## Data Availability

Not applicable.

## Abbreviations

AC: Adjuvant chemotherapy
ADC: Antibody–drug conjugate
AJCC: American Joint Committee on Cancer
AKT: Protein kinase B
B7-H3: B7 homolog 3
BART miRNAs: BamHI-A rightward transcript microRNAs
CAIX: Carbonic anhydrase IX
CAR-T: Chimeric antigen receptor T cell
CCRT: Concurrent chemoradiotherapy
CI: Confidence interval
CSC: Cancer stem cell
CT: Computed tomography
CTAR: C-terminal activation region
CTLs: Cytotoxic T lymphocytes
DSBs: DNA double-strand breaks
EBERs: EBV-encoded small RNAs
EBNA1: Epstein–Barr nuclear antigen 1
EBV: Epstein–Barr virus
EGFR: Epidermal growth factor receptor
EMT: Epithelial–mesenchymal transition
ERK: Extracellular signal-regulated kinase
FDA: Food and Drug Administration
FDG: Fluorodeoxyglucose
GP: Gemcitabine plus cisplatin
GSK3β: Glycogen synthase kinase 3 beta
GTV: Gross tumor volume
GWAS: Genome-wide association study
HIF-1α: Hypoxia-inducible factor 1 alpha
HLA: Human leukocyte antigen
IC: Induction chemotherapy
IKK: Inhibitor of κB kinase
IL: Interleukin
IMRT: Intensity-modulated radiotherapy
JAK/STAT: Janus kinase/signal transducer and activator of transcription
JAK2: Janus kinase 2
JNK: C-Jun N-terminal kinase
LDH: Lactate dehydrogenase
LMP1: Latent membrane protein 1
LMP2A: Latent membrane protein 2A
LOH: Loss of heterozygosity
MAPK: Mitogen-activated protein kinase
MAT2A: Methionine adenosyltransferase 2A
MDSCs: Myeloid-derived suppressor cells
MEK: Mitogen-activated protein kinase kinase
MHC: Major histocompatibility complex
MMP: Matrix metalloproteinase
MRI: Magnetic resonance imaging
mTOR: Mechanistic target of rapamycin
MTA1: Metastasis-associated protein 1
NCT: National Clinical Trial
NF-κB: Nuclear factor kappa B
NLR: Neutrophil-to-lymphocyte ratio
NPC: Nasopharyngeal carcinoma
OR: Odds ratio
PD-1: Programmed cell death protein 1
PD-L1: Programmed death-ligand 1
PET/CT: Positron emission tomography/computed tomography
PI3K: Phosphoinositide 3-kinase
RAF: Rapidly accelerated fibrosarcoma kinase
RAS: Rat sarcoma viral oncogene homolog
R/M: Recurrent or metastatic
RT: Radiotherapy
STAT3: Signal transducer and activator of transcription 3
TCR-T: T-cell receptor-engineered T cell
TGF-β: Transforming growth factor beta
TNM: Tumor–node–metastasis
TME: Tumor microenvironment
TPF: Docetaxel, cisplatin and fluorouracil
Treg: Regulatory T cell
UICC: Union for International Cancer Control
UTR: Untranslated region
VEGF: Vascular endothelial growth factor
VEGFR: Vascular endothelial growth factor receptor
WES: Whole-exome sequencing
WGS: Whole-genome sequencing
WHO: World Health Organization
